# Supplementary material for: Infodemiology of RSV in Italy (2017–2022): An Alternative Option for the Surveillance of Incident Cases in Pediatric Age?
Source: Children (Basel). 2022 Dec 16;9(12):1984. doi: 10.3390/children9121984 (PMC9777371; doi:10.3390/children9121984)
Supplement: Supplementary file 1 [file children-09-01984-s001.zip › children-2050770-supplementary.pdf]

Supplementary Table S1. Comparison in search volumes before and after December 2019.

Research Field	Up to December 2019 (Average ± SD)	After December 2019 (Average ± SD)	Student's t test for unpaired data with Welch correction (p value)
Google Trends™	Relative Search Volumes		
Bronchiolitis	14.40 ± 8.44	14.68 ± 20.83	0.915
RSV	3.13 ± 1.19	4.26 ± 11.11	0.377
Respiratory Syncytial Virus	2.03 ± 3.70	5.28 ± 14.00	0.052
Bronchitis	46.42 ± 15.44	34.73 ± 18.23	< 0.001
Pneumonia	8.35 ± 2.38	17.15 ± 15.72	< 0.001
Fever	26.82 ± 9.14	34.51 ± 12.58	< 0.001
Cough	44.10 ± 12.13	44.10 ± 12.13	0.540
Sneezing	19.03 ± 6.32	36.90 ± 14.20	< 0.001
Wheezing	13.60 ± 17.29	10.94 ± 12.16	0.288
Decrease in appetite	28.87 ± 9.21	32.40 ± 10.72	0.042
Respiratory Failure	28.26 ± 11.52	31.78 ± 17.30	0.152
Runny Nose	10.81 ± 5.65	27.24 ± 14.03	< 0.001
Common Cold	24.23 ± 5.06	30.53 ± 16.48	0.002
Influenza	14.66 ± 10.98	17.32 ± 18.14	0.286
Wikipedia	Daily Visualizations		
Bronchiolitis	90.70 ± 60.64	49.20 ± 65.99	< 0.001
Influenza	301.46 ± 140.82	840.92 ± 1559.29	0.003
Respiratory Syncytial Virus	33.87 ± 11.17	132.22 ± 322.68	0.009
Bronchitis	303.39 ± 146.57	104.24 ± 107.17	< 0.001
Pneumonia	601.18 ± 160.26	668.74 ± 974.86	0.549

Supplementary Table S2. Outline of variables included in Regression Analysis.

Variables	Model 1	Model 2	Model 3
ILI, incidence rates (outcome variable)	Total	0-4 years	5-14 years
Google Trends™ (explanatory variables)			
Bronchiolitis	YES	YES	YES
RSV	NO	NO	NO
Respiratory Syncytial Virus	YES	YES	YES
Bronchitis	YES	YES	YES
Pneumonia	YES	NO	YES
Fever	YES	YES	YES

Cough	YES	YES	YES
Sneezing	NO	NO	NO
Wheezing	NO	NO	NO
Decrease in appetite	YES	YES	YES
Respiratory Failure	NO	NO	NO
Running Nose	NO	NO	NO
Common Cold	YES	YES	YES
Influenza	YES	YES	YES
Wikipedia			
Bronchiolitis	YES	YES	YES
Influenza	YES	YES	YES
Respiratory Syncytial Virus	YES	YES	YES
Bronchitis	YES	NO	YES
Pneumonia	YES	NO	NO

Supplementary Table S3 Summary of regression analysis models.

Model	R	R square	SE	F	P value
**1**	0.867	0.752	1.83	27.113	< 0.001
**2**	0.851	0.725	3.51	25.526	< 0.001
**3**	0.804	0.646	6.44	21.216	< 0.001

Note: SE = Standard error of the estimate
